# Update on Transcatheter Interventions in Adults with Congenital Heart Disease

**DOI:** 10.3390/jcm13133685

**Published:** 2024-06-25

**Authors:** Angela Li, Jamil A. Aboulhosn

**Affiliations:** Division of Cardiology, UCLA Medical Center, Los Angeles, CA 90095, USA; jaboulhosn@mednet.ucla.edu

**Keywords:** adult congenital heart disease, transcatheter interventions, transcatheter valve replacement

## Abstract

The field of adult congenital interventions is unique in the world of cardiac catheterization, combining the structural concepts commonly employed in pediatric heart disease and applying them to adult patients, who are more amenable to device intervention as they no longer experience somatic growth. Rapid advances in the field have been made to match the growing population of adult patients with congenital heart disease, which currently surpasses the number of pediatric patients born with congenital heart disease. Many congenital defects, which once required surgical intervention or reoperation, can now be addressed via the transcatheter approach, minimizing the morbidity and mortality often encountered within adult congenital surgeries. In this paper, we aim to provide a focused review of the more common procedures that are utilized for the treatment of adult congenital heart disease patients in the catheterization laboratory, as examples of current practices in the United States, as well as emerging concepts and devices awaiting approval in the future.

## 1. Introduction

Over the years, the number of patients with adult congenital heart disease (ACHD) has continued to grow, and 90% of children born with congenital heart disease (CHD) are now expected to survive into adulthood [1]. In fact, the number of adult patients with CHD now far exceeds the number of pediatric patients, comprising two-thirds of all cases [2]. In parallel to the continued increase in the number of ACHD patients is an increase in the demand for non-surgical transcatheter interventions in an effort to avoid the risks inherent to open heart surgery and specifically the risks associated with reoperation. Transcatheter ACHD interventions are performed through minimally invasive approaches without the need for cardiopulmonary bypass, hence often decreasing the short term risks of major operative complications and significantly shortening post-procedural hospital stays [3]. In a retrospective review of catheterization cases within a dedicated ACHD center in Italy, the number of diagnostic and interventional cases both grew in proportion to the number of ACHD patients treated over the years included in the study [4]. To accommodate the unique complexity of these patients, there has been an increase in the breadth and complexity of interventional procedures as more innovations and devices have become available, such that the total number of possible interventions for CHD now exceeds fifty and will very likely continue to grow [1]. However, a comprehensive review of all the transcatheter procedures performed in ACHD treatment is beyond the scope of one manuscript. In this review, we aim to provide an update on a handful of procedures and devices that have been utilized for the treatment of ACHD patients in the catheterization laboratory as examples of the current practices within this growing field of medicine. We will focus primarily on devices utilized in the United States that we have a depth of experience with in our clinical practice in Los Angeles.

## 2. Ostium Secundum ASD Closure

Atrial septal defects (ASDs) account for 7–10% of all CHD, the most common of which is the ostium secundum defect [5]. ASDs may go undiagnosed during childhood and adolescence but often come to attention in adulthood due to the development of symptoms, which often include symptomatic arrhythmias and/or reductions in exertional capacity. ASDs make up between 20 and 40% of CHD conditions that are diagnosed in adulthood [6]. Patients with symptoms secondary to hemodynamically significant ASD and those who are asymptomatic but have evidence of right heart volume overload should be considered for defect closure, which is often associated with improved functional capacity and favorable cardiac remodeling [7,8]. A reported 80% of ostium secundum ASD cases are amenable to transcatheter closure [9] which has long been studied in the literature, with the “double disk” technique first being reported in a case report by Mills and King in 1976 [10]. It has since become favored over surgical closure, especially in older adults with multiple comorbidities, due to equivalent long-term outcomes with lower morbidity, shorter hospital stays, and improved cost effectiveness [11].

**Figure 1:**
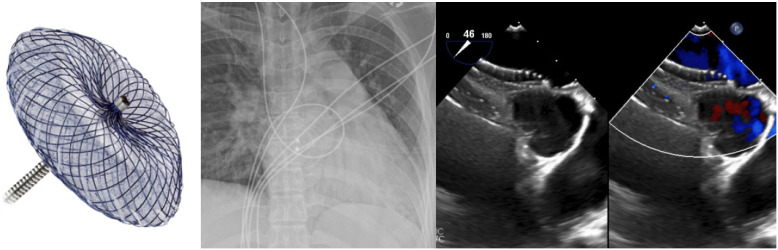
Amplatzer ™ Septal Occluder, seen in situ with 34 mm device placement.

Transcatheter ASD closure using nitinol-based devices became widely utilized in the late 1990’s and early 2000’s and over the past two decades has been shown to be safe, with short- and long-term mortality rates reported to be 0.01% and 0.1%, respectively [12]. The technical success rate is quoted to be around 96–98% [9], with the most studied device being the self-expanding nitinol-based double-disk Amplatzer Septal Occluder (Abbott, Green Oaks, IL, USA). The change in delivery system to the Amplatzer Trevisio delivery cable, which is composed of a stainless steel-coated nitinol core, now allows for a smoother delivery and increased flexibility during the release of the device [13]. With a wide range of sizes being available, it can close defects as large as 38 mm in diameter [5] (Figure 1), with the caveat that it has a known small but serious risk of cardiac erosion at 0.1% [12]. In a recent case-control study, risk factors for erosion were found to be deficiency of any rim and a large discrepancy between balloon sized and static ASD diameter [13], which may increase the risk of shearing the atrial free wall with the edge of a predominantly metallic device [12]. Techniques to try to overcome this risk include use of transesophageal echocardiogram (TEE) for imaging guidance [14] and avoiding oversizing the device by using a “stop-flow” technique during balloon sizing [12].

**Figure 2:**
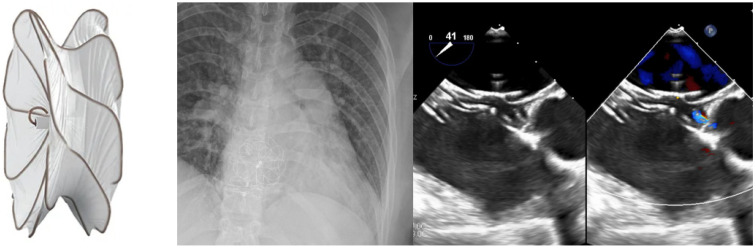
GORE^®^ CARDIOFORM ASD Occluder, seen in situ with simultaneous 32 mm and 48 mm device placement.

The GORE Cardioform ASD Occluder (WL Gore & Associates, Newark, DE, USA) is also a promising contender, as it is a softer device with a nitinol frame and a self-adjusting inter-disk waist, and the ability to close defects up to 35 mm in diameter [15] (Figure 2). It is released via a long delivery catheter containing a locking system and a retrieval cord, which allows for higher anatomical compliance during release [16]. A recent case series demonstrated safe and effective deployment of the Gore ASD Occluder device, including in challenging patients with large defect sizes or multiple deficient rims [15]. The newest Occlutech ASD Occluder (Occlutech, Helsingborg, Sweden), which received United States Food and Drug Administration (FDA) approval in January of 2024, is presented as being more flexible and less traumatic compared to the Amplatzer Septal Occluder due to having a reduced nitinol content in the left atrial (LA) disk, with size options up to 40 mm [14].

## 3. Secundum ASD Device Fenestration/Atrial Flow Regulator for Elevated Left Atrial Pressure

**Figure 3:**
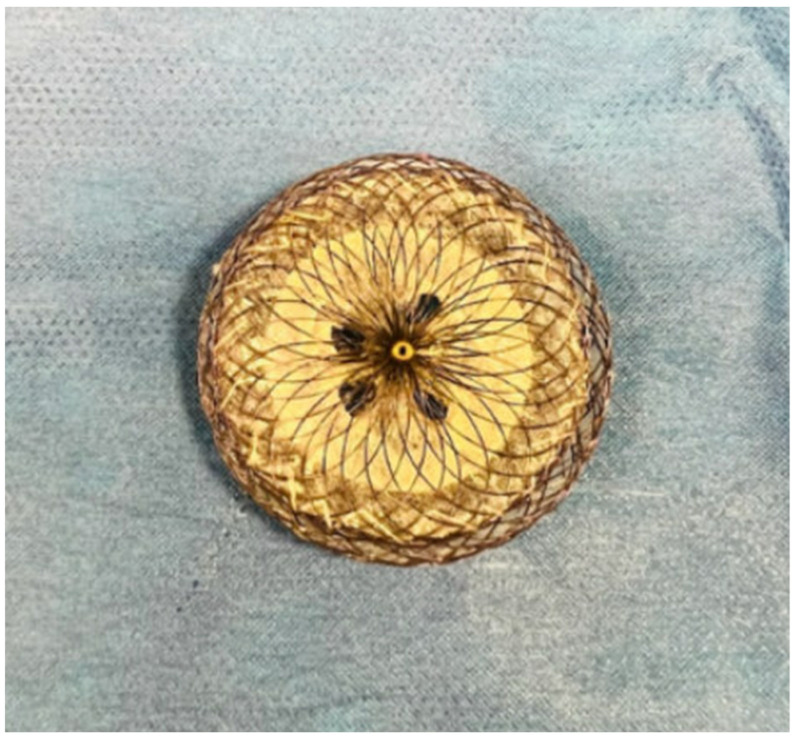
A 28 mm Amplatzer ™ Septal Occluder device with 4 × 2 mm self-fabricated fenestrations.

Complete closure of ASDs in the elderly population has been controversial, as over time, septal defects may provide decompressive atrial communication for interval development of left-sided diastolic heart failure or right-sided ventricular dysfunction and pulmonary hypertension [17,18]. An observational study previously demonstrated that complete closure of ASDs in patients with pre-existing elevated left atrial (LA) pressure would increase left ventricular (LV) preload, exacerbate diastolic dysfunction, and lead to pulmonary edema and heart failure [19]. It has now become customary to measure LA pressure during balloon test occlusion and consider the fenestration of ASD devices if there is a considerable increase, which has varied across studies as a rise in pressure of 3–10 mmHg [17,20,21]. Of the reported cases, short- and long-term follow up of patients with fenestrated devices demonstrated no adverse events, including no heart failure decompensation, although the fenestration did not always remain open on interval echocardiogram [17,21]. From the available case studies, it appears that fenestrations are generally self-fabricated with Amplatzer Septal Occluder devices (Figure 3) without a standardized method of creation [17,18,20,21].

The Atrial Flow Regulator (Occlutech, Helsingborg, Sweden) is a self-expanding, Nitinol-based double-disk device with central fenestration [22] that has been FDA approved for use in patients with severe symptomatic systolic or diastolic heart failure, as a controlled way to decompress the LA [23]. It has also been used off-label in CHD patients to create and reduce Fontan fenestrations [24], which are ideal considering its innate ability to provide a predictable shunt [25]. In this aspect, there could potentially be a role for its use as an ASD closure device for patients with elevated LA pressure, although the limited options in size (currently packaged with 21 or 23 mm discs) may prohibit its use in most patients with ostium secundum ASDs [22].

## 4. Superior Sinus Venosus ASD with Partial Anomalous Pulmonary Venous Connection-Covered Stenting of the Superior Vena Cava

The superior sinus venosus atrial septal defect (SVASD) makes up less than 10% of all ASDs [26] and is formed as the result of an embryological deficiency in the infolding of the atrial septum [27], wherein there is no posterior wall of the right superior vena cava (SVC) and no anterior wall of the right upper pulmonary vein (RUPV) [26]. The resultant association between the right SVC overriding the atrial septum and anomalous RUPV draining into the right atrial–SVC junction occurs in 90% of cases [28]. Due to the posterior location of the defect, diagnosis via transthoracic echocardiogram (TTE) is challenging and may prevent it from being identified until patients are symptomatic in adulthood [29]. Indications for closure are similar to other ASDs, including right heart volume overload, significant left to right shunt, and/or paradoxical thromboembolism [29].

Surgical correction has been the standard of care and was the only option until a novel transcatheter approach of placing a covered stent from the SVC into the right atrium was presented by Abdullah et al. at the Frankfurt CSI conference in 2013 [26] and first published by Garg et al. as a case report in 2014 [28]. The covered stent delineates the posterior wall of the SVC from the anterior wall of the RUPV, simultaneously closing the atrial defect while redirecting flow from the RUPV to the LA [28]. Since then, many heart centers have reproduced and refined this technique, with success rates of up to 97% [27]. Compared to surgical outcomes, follow ups for catheter-based interventions have shown similar reductions in right ventricular size and clinical symptoms [30,31], without the associated 6% risk of sinus node dysfunction [24,27,29,30]. In addition, a retrospective comparison between similar surgical and transcatheter groups demonstrated reduced lengths of hospital stay and lower rates of short term complications [27].

**Figure 4:**
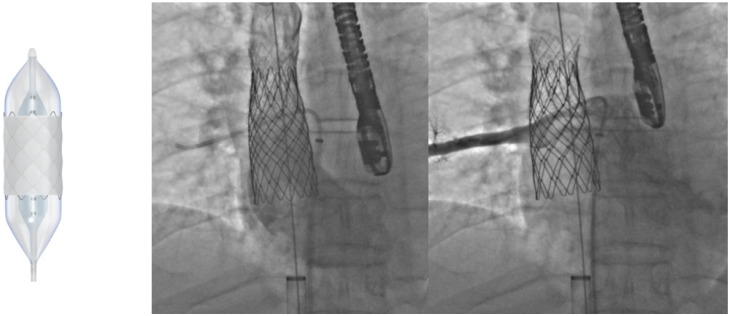
Covered CP Stent™, seen in situ with closure of superior sinus venosus ASD (**left**) while redirecting flow from RUPV to LA (**right**).

Contraindications to a transcatheter approach would be the presence of large pulmonary veins that drain exclusively into the SVC without a posterior wall connection to the left atrium [29], or RUPV obstruction after placement and dilation of the covered stent. This can be avoided with ex vivo feasibility planning via 3D models and cross-sectional imaging [30,31] in addition to in vivo testing of pulmonary vein patency via angiography and gradients on TEE [31]. Using balloon-expandable covered stents such as the 10-zig covered Cheatham Platinum (CP) stents (NuMED, Hopkinton, NY, USA) (Figure 4) instead of self-expanding stent grafts may also prevent unwanted pulmonary vein compression [31]. Through meticulous preparation, one larger case series of 25 patients determined that >75% of patients might be suitable for the transcatheter approach [31]. One major complication that has been documented is stent migration while flaring the caudal portion of the stent [31]. This has been addressed by placing additional stents cranially to “anchor” the covered stent, a technique that has been required up to 52% of the time in previous cases [31,32]. Future developments in stent design and delivery techniques may continue to advance this promising transcatheter alternative to the previous solely surgical correction for SVASD.

## 5. VSD Closure

Ventricular septal defects (VSDs) are the most common type of CHD with an incidence in 30% of all cases, including up to 10% of those diagnosed in adults [33]. In adults, VSD closure is indicated for hemodynamically significant left to right shunts (Qp:Qs > 1.5:1) [34] in cases of worsening aortic regurgitation (AR) [35] or with recurrent infective endocarditis [36]. Defects can occur anywhere along the septum, and more than 80% are perimembranous VSD (pmVSD) [36]. The closure of these defects poses an additional challenge due to their close proximity to conduction tissue and the aortic and tricuspid valves [37].

Surgical closure remains the gold standard for VSDs, but is associated with morbidity and mortality due to the need for cardiopulmonary bypass and sternotomy [38]. The first published case of the transcatheter closure of a VSD was by Lock et al. in 1988 with the Rashkind double umbrella device, which was used for both muscular and pmVSDs with varying degrees of success [39]. Since then, the Amplatzer Muscular VSD Occluder (Abbott, Green Oaks, IL, USA) has gained FDA approval for use in muscular VSDs only, after extensive publications with low complication rates [40,41].

The Amplatzer Membranous VSD Occluder (Abbott, Green Oaks, IL, USA) for pmVSD was introduced in the early 2000s, with device waist diameters ranging in size from 4 to 18 mm [42] and an asymmetric design of the left atrial disc. This was especially useful when defects were a short distance from the aortic valve [40] to prevent potential encroachment of the valve and iatrogenic AR [35]. However, initial studies reported a high rate of complete heart block (CHB), reaching up to 5.7% in some cases [43]. Subsequent studies have found that the majority of AV blocks are transient and improve with steroids [44], and the risk for CHB varies with age at time of procedure [33]. This has been attributed to the larger proportion of device area to ventricular septum size in children, which can compound the conduction system interaction caused by an oversized device [36]. In an analysis of pmVSD closures through the European Registry, all patients that required pacemaker but one were children [38], portending favorable outcomes for these procedures in adults.

**Figure 5:**
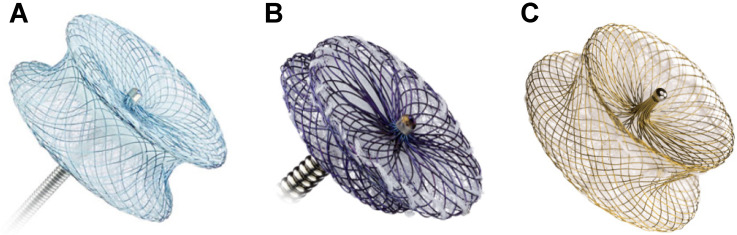
Amplatzer ™ Muscular VSD Occluder (**A**), Amplatzer ™ Membranous VSD Occluder (**B**), Occlutech PmVSD Occluder (**C**).

Device advances in recent years have further improved outcomes for transcatheter pmVSD closure. A new iteration of the Amplatzer Membranous VSD Occluder offers a reduction in radial force and increased stability, with thinner Nitinol wires making it a softer device. The Amplatzer Membranous VSD Occluder II has been used in case reports with no reported conduction abnormalities [45]. The Shanghai pmVSD occluder (Shape Memory Alloy Ltd., Shanghai, China) [46], the Occlutech pmVSD Occluder (Figure 5) (Occlutech, Helsingborg, Sweden) [44], and the KONAR-MF™ VSD occluder (Lifetech, Shenzhen, China) [37,47] are all pmVSD devices that are on the horizon which have been approved for use abroad and demonstrate promising results—a recent meta-analysis cited a pooled rate of overall success at 97.8%, a pooled incidence rate of AR at 2.0%, and a pooled incidence rate of CHB at 1.1%, comparable to the less than 1% risk for CHB in surgical VSD patients [44].

## 6. Transcatheter Pulmonary Valve Replacement (TCPVR)

It has been reported that of all newborns born with CHD, 20% of them have abnormalities of the right ventricular outflow tract (RVOT) [48]. They can then be separated by surgical interventions received: valvuloplasty or transannular patch which retain a “native” RVOT (tetralogy of Fallot, pulmonic stenosis) versus valved conduits or homograft replacements (transposition of great arteries, truncus arteriosus, Ross procedure) [49]. The eventual quandary for these patients as they progress to adulthood is when to pursue surgical reintervention, which has historically been a fine balance between allowing for a certain degree of pulmonary regurgitation (PR) and/or pulmonary stenosis (PS), at the expense of facing irreversible RV dilation and dysfunction [49]. The first transcatheter valve in a human was placed in the pulmonic position by Bonhoeffer et al. in 2000 [50]. This has since transformed the timeline for dysfunctional RVOT management, heralding a new era of TCPVR, which has become one of the most common procedures for patients with ACHD [51].

Bonhoeffer’s initial valve, a valved bovine internal jugular vein sewn inside a platinum stent [50], was eventually developed into the Melody valve (Medtronic, Minneapolis, MN, USA) and became commercially available in 2006, earning FDA approval in 2010 [48]. The Edwards SAPIEN/SAPIEN XT valve (Edwards Lifesciences, Irvine, CA, USA), initially designed for the aortic position, also became widely adopted for pulmonic use after favorable results from the COMPASSION trial [52], and has had specific FDA indications since 2016 [51]. Complications in TCPVR such as stent fracture, conduit rupture, coronary artery/aortic root compression [53], and valve embolization are known, but overall mortality is rare at 0.9% [49]. A retrospective cohort study and a meta-analysis separately compared outcomes between surgery and TCPVR, and found a significant reduction in length of hospital stay [54,55] and procedure-related complications [54] in TCPVR patients. During medium term follow up, both cohorts had similar valve hemodynamics; however, a higher rate of endocarditis was observed in the transcatheter group [54].

**Figure 6:**
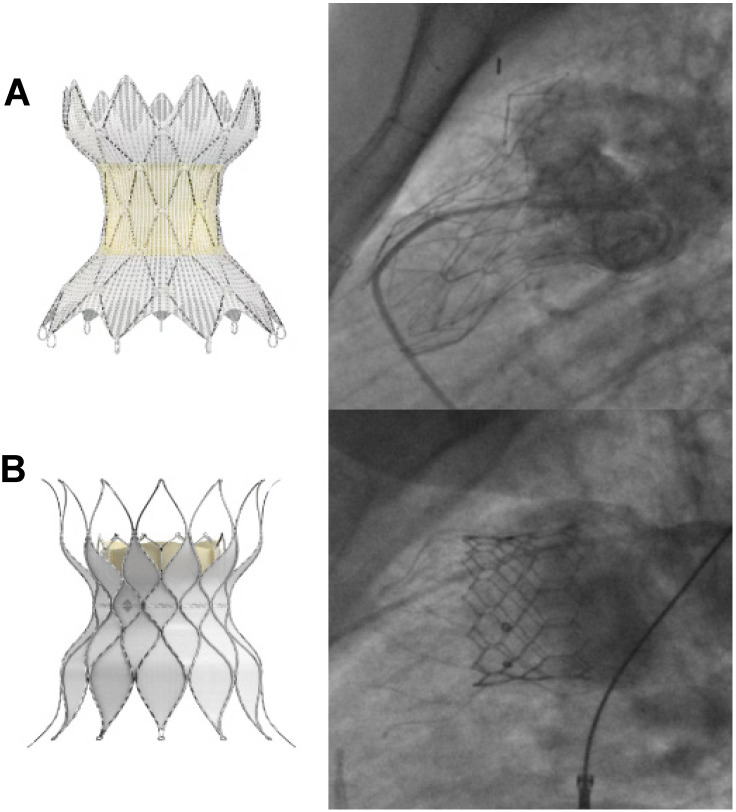
RVOT reducers: Medtronic Harmony TPV (**A**); Edwards Alterra Prestent with SAPIEN 3 (**B**).

TCPVR was initially only used to address surgical RVOT, but has now been extended to those patients with “native” RVOT [49]. Of the balloon-expandable valves, SAPIEN valves were preferred for use over Melody for their inherently larger diameters, but a hybrid surgical approach would sometimes be necessary to first place a RVOT ring off pump [49]. To address this, the Harmony TPV (Medtronic, Minneapolis, MN, USA) and the Alterra Adaptive Prestent (Edwards Lifesciences, Irvine, CA, USA) have been designed as transcatheter RVOT reducers (Figure 6). Newly FDA approved, these self-expanding devices can be deployed securely in a dilated RVOT anatomy without distorting or compressing nearby structures, and feature accommodations of inflow diameters up to 54 mm and 40 mm, respectively [56,57]. Looking abroad, the Venus *p* valve (Venus MedTech, Shanghai, China) is another self-expanding valve designed specifically for the dilated RVOT [58] and offers sizes up to a maximum diameter of 34 mm [59]. Technical success is quoted at around 97%, comparable to the rates of Melody and SAPIEN valves [58], and investigative trials of the Venus *p* valve have recently received approval from the FDA in the United States. Meanwhile the self-expandable Pulsta valve (TaeWoong Medical Co., Gimpo-si, Gyeonggi-do, Republic of Korea) has completed a multi-center clinical trial in South Korea and is undergoing Conformité Européenne (CE) approval studies in Europe [60].

**Figure 7:**
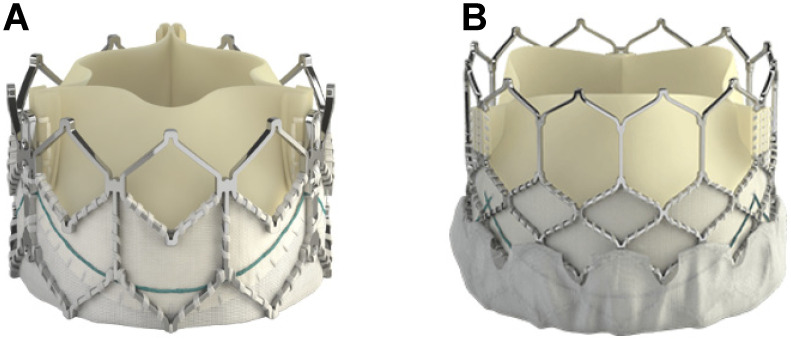
Evolution of Edwards SAPIEN valve: SAPIEN XT (**A**); SAPIEN 3 (**B**).

Newer devices and techniques are also being introduced to reduce periprocedural complications during TCPVR. The Edwards SAPIEN 3, evolved from the SAPIEN XT (Figure 7), boasts a “skirt” that minimizes paravalvular leaks [61], and has a higher radial strength which translates to lower incidences of stent fracture [51]. This precludes the need for presenting [62], a step that is typically required for Melody valves. Longer Dryseal sheaths have mitigated complications of tricuspid valve injury during valve advancement [63]. Finally, there have been attempts to address the risk of conduit tear in highly calcified RVOT conduits, with case reports noting successful lesion modification via the novel use of intravascular lithotripsy [64,65].

## 7. Coarctation of Aorta Stenting

Coarctation of the aorta (CoA) occurs in up to 0.04% of all live births [66] and is the fourth most common form of CHD, and is often associated with other intracardiac lesions such as bicuspid aortic valve [67]. The natural history of CoA has been linked to poor outcomes [68], with increased morbidity and reduced life expectancy [66]. In fact, the mean age of death without treatment has been quoted at 32 years of age [67]. Traditionally, indications for repair of CoA include gradients from the upper to lower extremities of more than 20 mmHg, excessive hypertension, and any degree of left ventricular dysfunction [67]. While initially a surgical intervention, the first successful balloon angioplasty of a coarctation was performed in a newborn in 1981 [67], and subsequently, the first endovascular stent placement was reported by O’Laughlin et al. in 1991 [69]. Since then, transcatheter intervention has superseded surgery and become the treatment of choice in adults [70]. Comparing surgical and transcatheter interventions, one study found that acute mortality rates were similar at less than 1–2% [70].

Balloon angioplasty has been associated with elastic recoil [71], and has a rate of restenosis quoted at around 7–36% [69]. There is also an increased risk of damaging the arterial wall, leading to aneurysm formation rates of up to 7.5% [70]. Currently, stent placement for CoA is favored over balloon angioplasty due to its sustained relief of obstruction [66] and greater decrease in coarctation gradient by providing adequate radial strength via reinforced stent structure [67]. Systematic review of outcomes post coarctation stenting have confirmed sustained lowering of systolic blood pressure [68]. A large study reviewing CoA stent outcomes over a 16 year period was very positive, with a 98.6% success rate compared to procedure-related death at only 0.3% [67]. Common complications recorded in a different review include stent migration (2.4%), aortic dissection (0.9%), and rupture (0.4%) [68].

**Figure 8:**
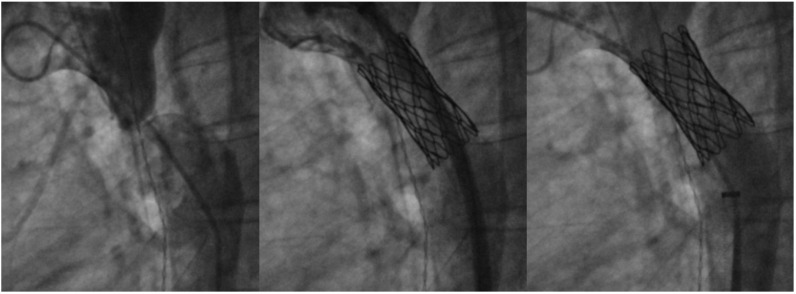
Stenting of a severe coarctation (**left**) with first a covered CP stent (**middle**) followed by a bare Palmaz XL 3110 stent (**right**).

Bare-metal stents have demonstrated incidence of aneurysm formation post procedure and are at increased risk for stent fracture [72]. Due to this, covered stents are now the preferred choice in coarctations with associated aneurysm, in older patients with increased risk for aortic injury [72], or if a more aggressive approach is needed in native CoA with tight stenosis. Balloon-expandable covered stents include the Cheatham-Platinum (CP) (NuMed, Hopkinton, NY, USA) and Palmaz Genesis (Cordis, Miami Lakes, FL, USA) stents (Figure 8), which are both closed-cell stents lined with polytetrafluoroethylene (PTFE) [72]. The CP stent is ideal for its zig pattern design, providing radial strength without significant foreshortening [71], and has distal tips which are less traumatic to friable arterial walls. Self-expanding stent grafts such as the Valiant^TM^ endoprosthesis (Medtronic, Minneapolis, MN, USA) may offer even less risk of trauma to the aorta but may not have optimal expansion in tight coarctations, and thus may be more suited for cases of aortic aneurysm without significant re-coarctation [72]. Concerns unique to covered stent platforms include the occlusion of side branches after placement, although it has been noted that occlusion of the left subclavian artery is usually well tolerated [72]. Outcomes with the use of covered stents remain promising; the COAST II trial for FDA approval demonstrated safety and efficacy of covered CP stent for aortic coarctation, with a reported freedom from surgical intervention at 100% during a 5 year follow up [73].

## 8. Collateral Coil Embolization

The Fontan procedure has been performed for more than 30 years for palliation of single ventricle physiology [74], using the basis of increased systemic venous pressure as the driving force to bring blood to the pulmonary vascular bed and bypassing the need for a ventricular pump [75]. When there is significantly increased pressure anywhere along this circulation, systemic venous to pulmonary venous collaterals can develop, which can be seen in up to one-third of Fontan patients [74,76]. It was thought that collaterals recanalize from embryologically preformed vessels [75] in response to increased pressure. To follow this anatomic correlation, supracardiac venous collaterals seem to form before the Fontan operation, while cardiac and infracardiac collaterals are more common after Fontan completion [74]. Coil embolization of veno-venous collaterals (VVCs) was initially proposed for progressive cyanosis [75] or hemodynamically significant shunts, with noted improvements in arterial saturations afterwards [74]. However, recent studies have argued against this practice, as elective occlusion of decompressing VVCs may lead to subsequently increased Fontan pressures and increased mortality [77].

**Figure 9:**
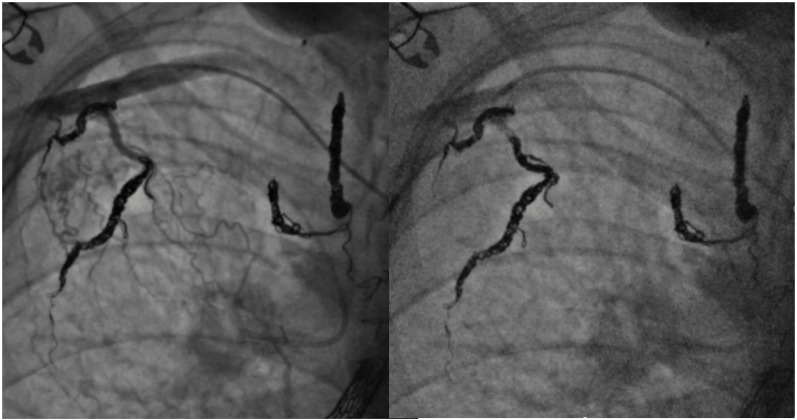
Coil embolization of previously coiled APCs with recanalization.

In contrast, aortopulmonary collaterals (APCs) are thought to develop secondary to chronic hypoxemia [78] or decreased pulsatility of flow in the pulmonary arteries [79]. Both occur in palliative single ventricles, and APCs are quoted to have a 20–30% incidence after the Fontan procedure [78]. As APCs bring blood from the aorta back to the pulmonary vascular bed, they are a source of considerable left to right shunt [80]. Hemodynamic assessment has shown that more than 40% of total pulmonary blood flow can originate from APCs [80], and as a result, can theoretically volume load the single ventricle and increase pulmonary artery pressures [81]. In children, a higher APC flow was associated with a longer postoperative hospital stay and prolonged duration of postoperative pleural effusion [80], and coil embolization may be associated with improving drainage of postoperative effusions [81]. Coil embolization of APCs has been reported since a large case series of 72 collateral vessels was carried out in 1989, with reported success rates of 96% for total or subtotal occlusion [82]. Certain centers propose APCs occlusion prior to any Fontan procedure, sometimes requiring multiple sessions to reduce the risk of heart failure post Fontan completion [78], and as a result, has shown low operative mortality [83]. However, evidence is often conflicting, as a study that quantitatively measured APC flow showed no effect on Fontan procedure outcome or higher Fontan pressure [81]. There is, however, a consensus that proper identification of APCs requires selective angiography over aortography [81], and that routine embolization of APCs prior to heart transplant can positively impact the amount of intraoperative bleeding encountered [77]. It should be noted that, in patients listed for heart transplant, collaterals should be coiled on an interval basis, as APC flow can increase within two months after coil embolization (Figure 9) [81].

## 9. Conclusions

The landscape of ACHD interventions is ever evolving, with a unique blend of devices, innovations, and skills being brought in from both the pediatric and adult interventional worlds to amalgamate into a field that is uniquely its own. As transcatheter ACHD technology continues to expand and improve, perhaps most encouraging of all has been the safety and tested durability of these interventions.

## Data Availability

No new data were created or analyzed in this study. Data sharing is not applicable to this article.
